# Exome-Wide Somatic Microsatellite Variation Is Altered in Cells with DNA Repair Deficiencies

**DOI:** 10.1371/journal.pone.0110263

**Published:** 2014-11-17

**Authors:** Zalman Vaksman, Natalie C. Fonville, Hongseok Tae, Harold R. Garner

**Affiliations:** 1 Virginia Bioinformatics Institute, Virginia Tech, Blacksburg, Virginia, 24061, United States of America; 2 Genomeon LLC, Floyd, Virginia, 24091, United States of America; The University of Hong Kong, Hong Kong

## Abstract

Microsatellites (MST), tandem repeats of 1–6 nucleotide motifs, are mutational hot-spots with a bias for insertions and deletions (INDELs) rather than single nucleotide polymorphisms (SNPs). The majority of MST instability studies are limited to a small number of loci, the Bethesda markers, which are only informative for a subset of colorectal cancers. In this paper we evaluate non-haplotype alleles present within next-gen sequencing data to evaluate somatic MST variation (SMV) within DNA repair proficient and DNA repair defective cell lines. We confirm that alleles present within next-gen data that do not contribute to the haplotype can be reliably quantified and utilized to evaluate the SMV without requiring comparisons of matched samples. We observed that SMV patterns found in DNA repair proficient cell lines without DNA repair defects, MCF10A, HEK293 and PD20 RV:D2, had consistent patterns among samples. Further, we were able to confirm that changes in SMV patterns in cell lines lacking functional BRCA2, FANCD2 and mismatch repair were consistent with the different pathways perturbed. Using this new exome sequencing analysis approach we show that DNA instability can be identified in a sample and that patterns of instability vary depending on the impaired DNA repair mechanism, and that genes harboring minor alleles are strongly associated with cancer pathways. The MST Minor Allele Caller used for this study is available at https://github.com/zalmanv/MST_minor_allele_caller.

## Introduction

Microsatellites (MSTs) are regions of repetitive DNA at which 1–6 nucleotides are tandemly repeated; and are present ubiquitously throughout the genome, both in gene and intergenic regions. Observations of somatic variation in MSTs have demonstrated that MST mutation rates are between 10 and 1000 time higher than that of surrounding DNA [1], [2], rendering microsatellites mutational “hot-spots” [3], [4]. The increased mutational rate of MSTs is thought to be primarily due DNA polymerase slippage and mis-alignment of the slipped structure due to local homology [5]–[7]. This difference in primary mutational mechanism suggests that, unlike non-repetitive DNA whose mutational spectrum is primarily SNPs, microsatellites are more prone to INDELs [4], [7], [8]. Specifically MSTs are prone to INDELs that are ‘in-phase’ or result in expansion or contraction by complete repeat units. For example, a dimer microsatellite will typically expand or contract by 2N nucleotides while a trimer will expand or contract by 3N [1].

MSTs are found in and around a significant number of coding and promoter regions and specific microsatellite variations have been linked to over 40 disorders, such as the CAG microsatellite whose expansion is associated with Huntington's disease and the CGG repeat whose expansion is associated with Fragile X [1], [9]. In addition, a more general increase in MST instability has been associated with colon cancer, which, if detected, results in better prognosis and can influence treatment [10], [11]. Currently, MST instability is clinically defined based on the results of a kit that tests somatic variation of 18–21 “susceptible” loci (PowerPlex 21, Promega). Although the test has been shown to be effective for identifying MST unstable colon cancer [12], it is significantly less effective for most other disorders including other cancers [13]–[15]. The ability to capture and discern variation patterns exome-wide would provide a more accurate and useful clinical data for a broader range of disorders. In recent reports next-gen sequencing has been used to uncover MST instability in intestinal and endometrial cancers by observing genotype changes in MSTs between tumor and healthy tissue [14], [15].

The goal of this research was to identify patterns of somatic variation in MSTs as a possible marker for genomic instability. We hypothesize that the variable nature of MSTs and the quantification of minor allele content makes them ideal candidates for in-depth next-gen analysis and that somatic variation of microsatellite loci can be quantified using high-depth sequencing. A broadening of the definition of MST instability to include changes in somatic variability and using an exome/genome-wide approach may enable a more accurate diagnosis of patients then what is currently provided by PowerPlex 21.

Somatic variability, novel genomic polymorphisms that arise within a cell population not found in the progenitors, plays a critical role in cellular reprogramming leading to the development and progression of cancer [16]. Suppression of mutations is essential for genomic stability, therefore cells have evolved multiple mechanisms to repair damaged or unpaired nucleotides [17], [18]. Currently the only established DNA repair defect that that has been directly linked to MST instability is mismatch repair (MMR). MMR impairments have been shown to increase somatic variation at MSTs in both cell lines and tumors [19]–[21]. Although the role other DNA repair mechanisms such as inter-strand crosslink repair (as seen in Fanconi anemia genes) and homologous recombination (HR) play in MST instability is less clear, both are important for genomic and chromosomal stability (reviewed by [22], [23]).

In this study we first show that we can robustly detect signatures of MST mutation bias and somatic variation occurring in cell lines in next-gen data including a high frequency of in-phase INDELs. We are then able to construct a pattern of somatic MST variation (SMV) by using DNA repair proficient cell lines. Our results indicate that ∼5% of microsatellite loci show somatic variation, i.e. have at least one additional non-haplotype allele present. Finally, we are able to differentiate between cell lines with known defects in various DNA repair mechanisms (mismatch repair, DNA crosslink repair, homologous recombination), which correlate with an altered distribution of loci with non-haplotype alleles. These findings suggest that signatures that distinctly define specific defective DNA repair mechanisms can be gleaned from next-gen sequencing data and that this information has the potential to be utilized for detection of individuals with altered levels of somatic variation that are at increased risk of disease or the evaluation of patient's tumor that may yield clinically actionable information.

## Methods

### Cells, DNA prep and sequencing

HEK (human embryonic kidney) and MCF10A (immortalized breast epithelial) and HEK293 (human embryonic kidney) cells were obtained from ATCC. PD20 and PD20 RV:D2 (FANCD2 and FANCD2 retrovirally corrected) cell lines were obtained from the Fanconi Anemia Foundation (Eugene OR). Sequencing data for Capan-1 cells was previously published by Barber and coworkers [12].

PD20, PD20 RV:D2 and HEK293 cells were grown at 37°C with 5% CO_2_, in DMEM supplemented with 10% FBS (Invitrogen) and 1X pen/strep (Invitrogen) to 80% confluence. MCF10A cells were grown to confluence in DMEM/F12 medium (Invitrogen, Carlsbad, CA), supplemented with 5% horse serum (Invitrogen), antibiotics- 1X Pen/Strep (Invitrogen), 20 ng/mL EGF (Peprotech, Rocky Hill, NJ), 0.5 mg/mL hydrocortisone (Sigma), 100 ng/mL cholera toxin (Sigma), and 10 µg/mL insulin (Sigma) at 37°C with 5% CO_2_. All cell lines were collected by trypsinazation and prepared for DNA extraction. DNA was extracted using the Qiagen DNAeasy kit (Qiagen) as per manufacturers instructions.

Since PD20 RV:D2 were derived from PD20 cells by retroviral insertion of the corrected FANCD2 gene we confirmed the maintenance of the corrected version using the sequencing data. Further, a comparison of growth-curves showed an order of magnitude more cells 48 hours after exposure to the DNA interstrand cross-linker Cisplatin, confirming a partial rescue phenotype.

### Sequencing and analysis pipeline

Exome paired-end libraries were prepared using the Agilent (Chicago, IL) SureSelectXT Human All Exon V4 capture library. 2×100 bp reads were obtained using an Illumina (San Diego, CA) HiSeq 2500 instrument in Rapid Run mode on a HiSeq Rapid v1 flowcell. Indexed reads were de-multiplexed with CASAVA v1.8.2.

Paired-end sequencing reads were trimmed using fastX_Toolkit and aligned to HG19/GRCh37 human reference genome (http://www.genome.ucsc.edu) using BWA-mem. The output was then sorted, indexed and PCR duplicates were removed using SAMTOOLS [24]. Bam files were then locally realigned and target loci marked using GATK IndelRealigner and TargetIntervals. MST alleles were retrieved and analyzed using software described in the next section.

### Microsatellite minor-allele software

A catalogue of MST loci was generated from the HG19/GRCh37 reference genome using Tandem Repeats Finder [25] (with the following parameters: 2.7.7.80.10.18.6). The list was filtered to remove any loci that were shorter than 8 nucleotides, had less than 3 copies of a given motif unit or were below 85% sequence purity. Duplicated loci were identified based on sequence purity and sequence length and were removed.

MSTs were analyzed using a custom MST minor-allele caller based on GenoTan and ReviSTER software [26], [27], which were developed by this group to improve MST haplotype predictions (https://github.com/zalmanv/MST_minor_allele_caller). The minor-allele caller extracts marked MSTs from bam files using SAMTOOLs. MST loci are called based on predicted alignments and an adjustable length flanking sequence (this study used either 5 or 7 nucleotide sequence). Reads with low base call scores (below a base score of 28) for nucleotides within the repeats and those with mapping quality score below 10% were eliminated. Alleles are initially called only when two or more reads, verified in both directions of a paired-end run, have the same sequence. All alleles for a given locus are binned with the number of supporting paired-end reads. The final number of alleles is computed based on a user specified minimal requirement of substantiating reads (for this study the minimum number of substantiating reads is either 2 or 3 reads per allele). If more than one allele per locus was found, zygosity and the sequence length difference from the most common allele were recorded. Heterozygotic loci were called using the following criteria as described and confirmed in the GenoTan and ReviSTER manuscripts [26], [27]: 1) it is the second most common allele, 2) The number of confirming reads is greater than 25% of the total reads for the locus or greater than 50% of the depth for the most common allele, if the total is below 25% of the total depth.

In addition to MST loci, we also generated a somatic variability profile for non-MST loci. To make the data comparable we randomly selected 3 million loci, each consisting of 15 nucleotides segments, from the HG19 genome. We then filtered out any loci that intersected with our MST and were left with over 2 million loci. The same pipeline as for MSTs was used to generate the data for non-MST loci. This data yielded information on the number of loci with minor alleles and type of mutation (SNPs and INDELs).

### Sequence validation and allele calls validated by independent Sanger sequencing method

The MST minor-allele caller we use in this paper is a modified version of a published and experimentally verified code, however to further validate the multi-allele capability of the modified code 30 loci, including 17 showing multiple alleles, were verified using Sanger sequencing. Figure S1A in file S1 shows the data from the minor-allele caller output at one of these loci, chr10:72639137-72639161, at which we would predict at least 3 alleles to be present in this sample (MCF10A) with lengths of 21, 23, and 25 nucleotides. Sanger sequencing confirmed that multiple alleles were present, with the alleles being greater than 21 nucleotides long (figure S1B in file S1). Of the 30 loci 28 loci verified the genotype and 14 of 17 loci with minor alleles also had visible minor alleles by Sanger sequencing.

### Modeling error rates to establish rules that differentiate errors from high confidence minor alleles

Two methods were used to generate models of NGS runs for chromosomes 17 and 21; 1) Wgsim (https://github.com/lh3/wgsim) a commonly used paired-end read generator and 2) in-house designed generator. Both methods were set to have a per nucleotide error rate between 0.5% and 5%. The major difference between the two methods was that wgsim was used to obtain modeling data with fairly similar coverage (read depth) across the reference chromosome while the lab-designed algorithm allowed for a more variable coverage as is observed in a typical next-gen sequencing run. The generated fastq files were run through the same pipeline as actual real sequencing data. The accuracy of the pipeline was analyzed by the verification of the predicted alignment. Predicted error rates ranged between 1.3% and 1.9%, with the majority of errors due to misalignments.

## Results

We modified a previously published and verified MST genotyper [26] to enumerate all possible alleles present within next-gen data, as opposed to only capturing the most common (haplotype) alleles. We first characterized the error which may cause false positive allele calls via a parametric sensitivity study conducted on *in-silico* generated data, and showed that our measure can then be used to accurately quantify minor alleles and thus be used to distinguish between mutational mechanisms that are exhibited in different cell lines. To accomplish this, we establish a baseline SMV profile from DNA repair proficient cell lines, and compared this to what is seen in cell lines with various DNA repair defects.

### Characterizing the effect of sequencing error on minority allele calling

**Figure 1:**
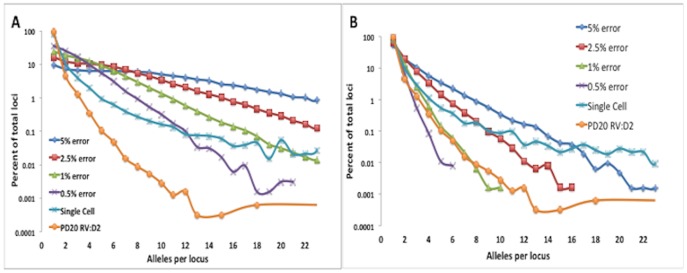
Effects of sequencing error and the minimum number of reads required to call an allele on the number of alleles called in sequencing data. Modeling data with different error frequencies (0.5%–5%) showed an increase in loci with multiple alleles as error increased when both 2 (A) and 3 (B) reads were minimally required to call an allele. In contrast, standard exome sequencing data from DNA repair proficient cells (PD20 RV:D2 cells) and exome sequencing after whole genome amplification from a single cell were insensitive to the cut-off used.

This analysis evaluates each MST locus to establish the one or two alleles that define the genotype, then it robustly calls additional non-haplotype or ‘minor’ alleles that are present at lower frequency within next-gen data. However, the accuracy of such minority allele calls can be significantly affected by sequencing errors found within the raw reads that map to each locus. To minimize the number of false positive ‘alleles’, we first established the minimal number of reads necessary for confirming an allele in the presence of typical next-gen errors. It has been established by a number of studies that 3 reads mapped to a loci is sufficient to properly call major alleles [28]–[30]. To corroborate this, we created an *in-silico* sequencing data set for chromosomes 21 and 17, with randomly generated errors ranging from 0.5% to 5% which mimicked next-gen sequencing data in both the error types that were created and read coverage per locus (results depicted in figure 1).

**Figure 2:**
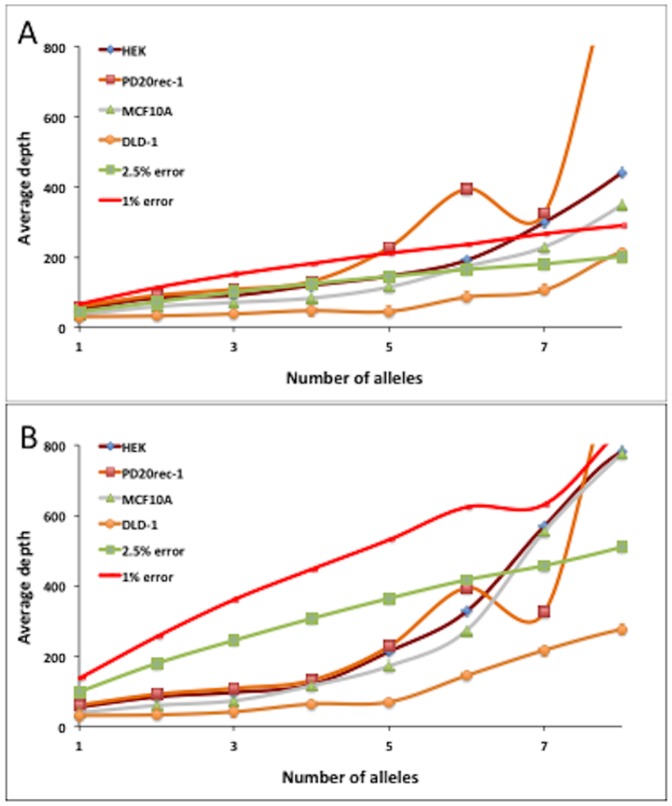
Variation in average depth per locus cannot explain the number of loci with minor alleles. The average read depth at loci with increasing numbers of alleles using A) 2 and B) 3 confirming reads per allele for in-silico generated data using 1% and 2.5% induced error rate for 4 different cell lines.

**Figure 3:**
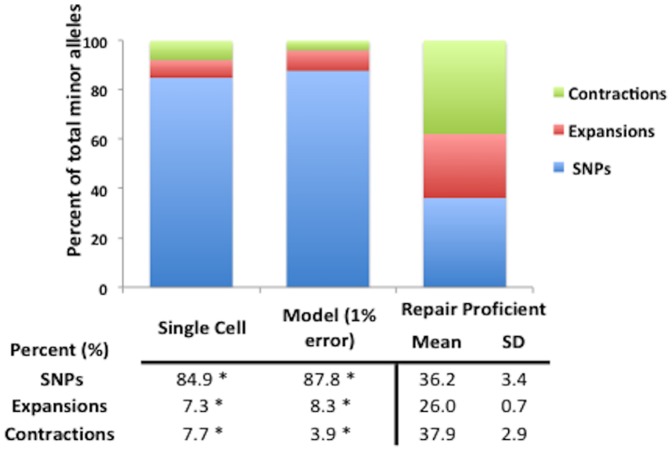
DNA repair proficient cells vary significantly from the *in-silico* modeling and single cell sequencing analysis with respect to SNPs and INDELs. The percent of SNPs, expansion and contractions for single cell sequencing and the *in-silico* model as well as the mean and standard deviation for the control cell lines. * significant difference p<0.01.

We first determined the parameters required to optimize the measurement of the fraction of loci *without* minor alleles in sequencing data with the above-mentioned error rates. Alignment and zygosity calling accuracy is displayed in table S1 in file S1. The sequencing data generator produced between 8 and 10.5 million reads that contained over 58,000 targeted MSTs. Over 98.5% of the reads mapped correctly with an accuracy of over 99.8% in coding regions (regions captured by exome sequencing). The accuracy of zygosity calls was over 99.98% for all error rates. Next we varied the minimum number of reads covering a locus required to call an allele. Changing the threshold from 2 confirming reads (figure 1A) to 3 confirming reads (figure 1B) statistically and significantly decreased the fraction of loci with more alleles than the haplotype number (1 if homozygotic or 2 if heterozygotic). Using a threshold of 2 confirming reads per allele, the fraction of loci without minor alleles identified (due to sequencing errors being interpreted as alleles) was 19–62% for simulated data sets with error rates ranging between 5%–0.5% respectively (figure 1A), indicating that requiring only 2 reads to identify an allele leads to a high level of false alleles. By increasing the threshold to 3 confirming reads the percent of loci without minor alleles increases to 73–99% for the same data set (figure 1B). By increasing to 4 confirming reads per allele we further increase the number of loci without minor alleles 87%–99% (figure S2A in file S1). However, at error rates close to the actual HiSeq rates (of ∼1%), we only saw a modest increase in the number of loci without minor alleles, a change from 97% (3 reads per allele) to 99% (4 reads per allele). This is in contrast to an increase from 61% with 2 reads per allele to 97% with 3 confirming reads per allele.

We next examined how sequencing error might affect the number of alleles present in our data. To do this we used modeling data with error rates similar to the actual HiSeq error rate (1%) and 2.5% error (figure 2), and determined the average read depth per locus with increasing alleles. For the *in-silico* generated data, we found a linear increase in the total read depth as the number of alleles increased (using 2–4 confirming reads per allele) up to 8 alleles (figures 2 and Figure S2 in File S1). A comparison of these results to actual sequencing data from our cell lines (discussed in more detail later) shows that when 3 or more reads are required to confirm an allele, the number of alleles called for a given read depth is greater than what would be expected from error, even at a rate of 2.5% which is substantially more than the observed next-gen error rate of 1% (figure 2B and Figure S2B in File S1), i.e. more alleles are called at a lower read depth in the actual data than would be present due to error. Based on these results, requiring a minimum of 3 reads covering a locus to confirm an allele minimizes the number of ‘false’ alleles being identified due to sequencing error.

### Polymerase slippage vs. nucleotide misincorperation

Another potential source of error in calling alleles from sequencing data is amplification errors induced during the library preparation process [31]. These errors would likely be present at higher frequency than errors generated during sequencing [31], [32]; therefore cannot be minimized by solely increasing the minimum read coverage (as above). Somatic mutation of MSTs is primarily associated with polymerase slippage [33], [34], which is thought to cause the characteristic INDEL bias [31], [35], [36]. In contrast, nucleotide mis-incorperation errors during *in-vitro* amplification would be predicted to lead primarily to SNPs in sequencing data [37]. Both of the mentioned DNA synthesis methods would lead to an increase in the number of loci with non-haplotype alleles, however with a predicted variation pattern that is distinctly different. To differentiate between the two predicted SMV patterns including minority alleles, and to assess the influence of nucleotide mis-incorporation/amplification error on our results, we compared a standard exome sequence from cells which are proficient for DNA repair (described later) that did not undergo whole genome amplification (WGA) with data from the sequencing of a single cell [38] which would be expected to have no somatic variation within the sample, but has necessarily undergone WGA to generate the quantity of DNA necessary for sequencing. Therefore, for the WGA sample, presumably all non-haplotype alleles present are due to amplification error. As expected, genome amplification increases the number of loci with non-haplotype alleles (figure 1) to 11.3% and 7% of the total with a threshold of 2 and 3 reads, respectively. The DNA repair proficient cells, which did not undergo extensive amplification, were only decreased by 1.7%, from 7% to 5.3%, by altering the minimum read cutoff. From this it can be concluded that neither errors during library prep nor during the sequencing run account for more than 4 percent of the total non-haplotype alleles detected.

Approximately 85% of mutations found within microsatellite loci in the WGA single-cell data were SNPs, which is expected as a consequence of polymerase errors during amplification. These results were comparable to those predicted by our model, which showed that ∼88% of the total minor alleles were composed of alleles carrying SNPs rather than INDELs (Figure 3). In contrast, SNPs account for only 36% (±3.4%) of the total minor alleles in DNA repair proficient cell lines. In addition, although for all the DNA repair proficient cell lines the most common MST motifs with minor alleles observed were mono-nucleotide repeats found within 56%–66% of loci, loci containing tri-nucleotide motifs accounted for over 55% of the total loci with minor alleles in the WGA data (table S2 in file S1). These results further support the hypothesis that this approach can differentiate between distinct MST mutational profiles: INDELs, particularly at mono-nucleotide runs predominantly reflect DNA repair proficient biological SMV whereas SNPs in MSTs, particularly at tri-nucleotide motif containing loci are predominantly amplification-induced errors or potentially due to altered DNA maintenance capacity. This is further supported by a similar study that has found that the majority of MSTs that are variable within the normal population (individuals sequenced as part of the 1,000 Genomes Project) are predominantly INDELs at mono-nucleotide runs [30].

### MST vs non-MST regions

MSTs are considered to be more susceptible to mutations than the surrounding non-repetitive DNA regions [3], [14], [39]. Because of this, one could expect that non-MST regions would have less somatic variability (non-MST equivalent of SMV) than MST regions. In order to perform a fair comparison with the MST data, 2 million segments consisting of 15 nucleotides each were randomly selected throughout the genome. The same analysis as was performed on loci containing MSTs was also applied to these non-MST regions. It was found that for these non-MST loci the average fraction of loci that were homozygotic was 98.9% with a standard deviation of 0.2, while only 96.7% of the MST containing loci was homozygotic. Even more significant, only 2% (standard deviation of 0.2) of the non-MST loci (homozygotic and heterozygotic) had minor alleles, while 5.1% of the MST loci harbored minor alleles (table 1). Further, a comparison of SNP and INDEL distributions indicated that, unlike MST regions where INDEL variations prevail (64%), SNPs account for the majority (96.9%) of the differences in minor alleles at non-MST loci (table 2). Taken together, these results confirm that, consistent with the literature, MSTs are more susceptible to mutation [2]–[4], [34].

**Table 1:** Exome sequencing data indicates that MST and non-MST haplotype and somatic polymorphism are reproducible in DNA repair proficient cell lines.

	Microsatellite loci				Repair Proficient		Non-Microsatellite loci				Repair Proficient	
**Percent (%)**	PD20 RV:D2-1	PD20 RV:D2-2	MCF10A	HEK293	Mean	SD	PD20 RV:D2-1	PD20 RV:D2-2	MCF10A	HEK293	Mean	SD
**Homo-zyg**	96.8	96.8	96.4	97.0	96.7	0.3	99.0	99.0	98.6	99.1	98.9	0.2
**Hetero-zyg**	3.2	3.2	3.6	3.0	3.3	0.3	1.0	1.0	1.4	0.9	1.1	0.2
**Multi-alleles**	5.4	5.3	4.5	5.3	5.1	0.4	1.7	2.0	2.1	2.1	2.0	0.2

**Table 2:** MST and non-MST containing loci from exome sequencing of DNA repair proficient cells, but not from sequencing of a single cell after whole genome amplification, show the expected high ratio of INDELs (expansions and contractions) to SNPs.

	Microsatellite loci				Repair Proficient		Non-microsatellite loci				Repair Proficient	
Percent (%)	PD20 RV:D2-1	PD20 RV:D2-2	MCF10A	HEK293	Mean	SD	PD20 RV:D2-1	PD20 RV:D2-2	MCF10A	HEK293	Mean	SD
SNPs	33.6	32.7	41.4	36.9	36.2	3.4	96.9	96.6	96.8	97.2	96.9	0.2
Expansions	26.2	27.0	25.3	25.5	26.0	0.7	1.3	1.6	1.6	1.4	1.5	0.1
Contractions	40.3	40.3	33.3	37.5	37.9	2.9	1.8	1.8	1.6	1.3	1.6	0.2

### Reproducibility within a cell line

The objective of this study is to characterize the pattern of SMV from DNA repair proficient cells and then compare to cell populations in which DNA repair is compromised. SMV changes associated with disease will likely be subtle and require highly reproducible control data. To test the reproducibility of SMV measurements within a cell line, two biological replicate cultures of PD20 RV:D2 (PD20 RV:D2-1 and PD20 RV:D2-2) cells were grown separately and sequenced. PD20 RV:D2 are fibroblasts derived from an individual with Fanconi Anemia subgroup D2, retroviraly complimented with a functional copy of FANCD2 [40]. Using a minimum read depth cutoff of 15 to genotype a given loci, we successfully called over 280 K and 250 K loci (at an average depth of 52 and 45 reads per locus) for PD20 RV:D2-1 and 2 respectively. Both samples showed a similar SNP to INDEL ratio, with INDELs making up over ∼67% of the minor alleles (table 2). A genotype analysis showed that approximately 96.8% of called loci were homozygous while heterozygosity was observed in ∼3.2% of the loci called (table 1). Comparison of those loci that were called in both samples shows that haplotype discordance (i.e. homo- or heterozygotic using standard genotyping) was 1.1% (table 3), of which 92% were due the fraction of reads supporting a second allele being below the haplotype threshold (see method) and was therefore counted as a minor allele instead of a second haplotype allele, as is the convention in established genotype callers. Only 173 discordant loci were due to sequence differences between the two samples.

**Table 3:** Percent concordance/discordance of haplotype and loci with minor alleles for cell lines.

	Genotype	More then haplotype alleles		Haplotype Allele number
	Discordance	Concordance	Discordance	Concordance
PD20 RV:D2-1 & -2	1.06	3.43	2.69	93.88
PD20rec-1 & PD20	1.15	2.50	3.07	94.43
PD20rec-1 & MCF10A	3.79	1.99	3.95	94.10
PD20rec-1 & Capan-1	2.68	1.92	12.68	85.40
MCF10A & Capan-1	2.19	1.24	13.62	85.10

**Figure 4:**
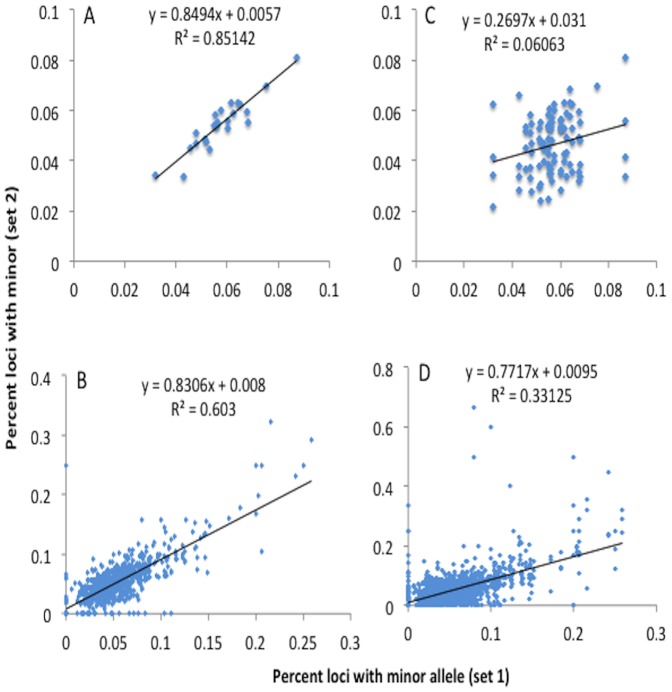
A regression analysis indicates a significant within and between cell line correlation in the fraction of loci with one or more minor alleles. Full factorial plots of the fraction of loci with minor alleles by chromosome, regression line and correlation coefficient for A) PD20 RV:D2-1 and 2 C) PD20 RV:D2-1, 2, MCF10A and HEK293. Also full factorial plots of the fraction of loci with minor alleles for the corresponding 1 million base segments of all the chromosomes, a regression line and the correlation coefficient for B) PD20 RV:D2-1 and 2 D) PD20 RV:D2-1, 2, MCF10A and HEK293.

**Figure 5:**
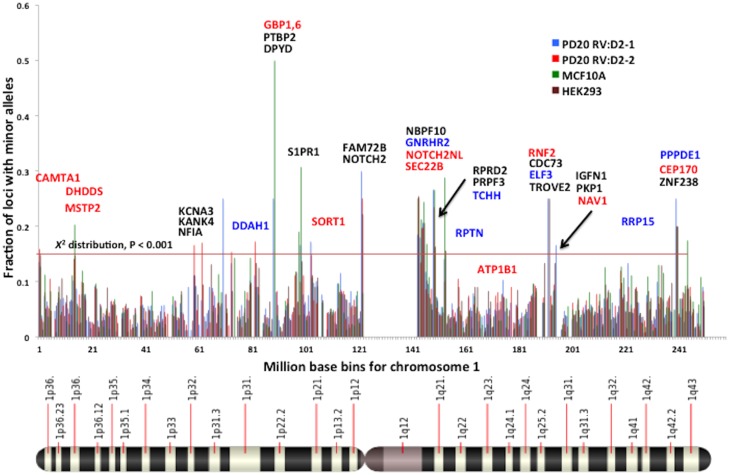
The distribution of MST loci showing somatic variability for chromosome 1 binned into 1 million base regions in PD20 and the derived PD20 RV:D2 cell line. The horizontal line demarcates outlier segments, based on a X^2^ distribution. All genes shown were found to contain exonal MSTs that with at least 2 minor alleles in both PD20 RV:D2 samples and were found in regions that exceeded the demarcated level. Genes shown in red were found to contain exonal MSTs with at least 2 minor alleles in all 4 DNA repair proficient cell line samples and those shown in blue were found in 3 of the 4 samples. The chromosome image shown at the bottom was obtained from http://en.wikipedia.org/wiki/Chromosome_1 _(human).

For the purpose of this study SMV is defined by the presence of variant MST alleles that are supported by a minimum of 3 confirming reads but do not contribute to haplotype. An analysis of variant MST alleles found a total of 5.4% and 5.3% of MST loci in the PD20 RV:D2-1 and 2 samples, respectively, had 1 or more minor alleles (table 1). The concordance of loci without minor alleles in either sample is 93.9% while 3.4% of loci have at least one minor allele in both samples. By concordance we mean a locus has minor alleles or the same haplotype in multiple samples. Conversely, discordance, where a locus in only one of the compared samples had minor alleles, was 2.7% (table 3). To confirm the significance of these values, we calculated the probabilities of concordance and discordance based on a cohort of randomly selected loci (5.4% and 5.3% of a total samples), which was <0.25% concordant, and compared with our results. Using a Pearson's goodness of fit X^2^, we verified that the concordant loci are not randomly distributed (p<0.0001). To determine within cell line reproducibility we compared the percent of loci having minor alleles by chromosome as a whole and binned into a million base regions. A linear regression model comparing the percent of loci with minor alleles for each chromosome (as depicted in figure S3 in file S1) shows a significant correlation (R^2^ = 0.85 and p<0.001) between two independently cultured samples (Figure 4A). Similarly, a comparison of the binned chromosome also shows a significant correlation (R^2^ = 0.60 and p<0.001, figure 4B). Visualization of the distribution of fraction of MST loci showing somatic variation in a representative chromosome (chr1), depicted in figure 5, indicates specific chromosomal regions that may harbor SMV “hot-spots”. An evaluation of MST loci in translated (exon) regions found over 820 genes containing MSTs with a minimum of 2 minor alleles in both PD20 RV:D2 samples, with some of genes found within segments of chromosome 1 with increased SMV depicted in figure 5 (a complete list of exonal MSTs with the minor alleles called, for all cell lines discussed in this paper are available in File S2).

Taken together these results support our hypothesis that this method truly reflects SMV rather than error generated during sequencing and that the results are highly reproducible. The data further suggests that within an individual or cell line, specific genomic regions may contain MSTs that are more susceptible to somatic variability.

### Reproducibility between cell lines

To begin to establish a SMV baseline for DNA repair proficient cells, we compared the haplotype, minor allele and SNP/INDEL distributions for two DNA repair proficient cell lines and the PD20 RV:D2 cells discussed above. MCF10A cells are immortalized breast epithelial cells derived from a healthy human female and HEK293 cells are a human embryonic kidney cell line derived from a healthy male fetus. Sequencing produced over 45 million reads with over 170 K microsatellite loci called at an average depth of 42 reads per locus for HEK293 cells and over 190 K microsatellite loci called at an average depth of 39 reads per locus for MCF10A cells. Considering major alleles only, 96.4% and 97.0% of all MST loci, respectively, are homozygotic (table 1). The average fraction of loci with minor alleles for all three cell lines was 5.1% with a standard deviation of 0.4%. Although MCF10A cells had fewer loci with minor alleles than the PD20 RV:D2 and HEK293 cells (4.5% compared with 5.3% and 5.4% respectively, table 1), and showed a difference in the fraction of secondary alleles with SNPs compare to INDELS (table 2), MCF10A was not considered an outlier (using Grubb's test for outliers). When we compared the haplotype and minor allele concordance between two non-related cell lines, MCF10A and PD20 RV:D2, we found that 3.8% of loci have different genotypes with only 60% due to haplotype differences. For those loci with minor alleles, discordance is 4.0% and concordance is only 2.0%, the result is significantly above what would be anticipated by chance with Pearson's X^2^ (i.e. <0.3%,). Interestingly, a full factorial comparison of the fraction of loci with minor alleles for each chromosome (as depicted in figure S4 in file S1), using a linear regression model, found a non-significant correlation (R^2^ = 0.061 and p<0.23, figure 4C). However, a correlation using the 1 million base bins is significant with an R^2^ value of 0.33 and a p<0.0001 (figure 4D), supporting the concept that certain regions contain minor allele susceptibility hot spots. These results demonstrate substantial reproducibility between unrelated independently grown DNA repair proficient cell lines even when the samples are derived from different tissues of origin. These results also suggest that a baseline profile of SMV can be established for DNA repair proficient cells to compare to cell lines with DNA repair defects.

### SMV in cells with compromised DNA repair capacity

Thus far we have established that (1) three DNA repair proficient cell lines show similar SMV with low variability both within and between cell lines and that (2) we can differentiate between different SMV trends based on the ratio of INDELs to SNPs. However, the larger goal of this study is to compare SMV patterns between cell lines representative of healthy individuals and those that may have altered DNA repair capacity. To test this, we evaluated 3 cell lines commonly used to study DNA repair and stability. DLD-1 cells are MST instability (MSI) high colon cancer cell line, impaired in Mismatch repair (MMR), selected as positive controls for this study [41]. Capan-1 cells were sequenced previously [12] and are a BRCA2- cell line that can propagate in culture. PD20 cells are from a FANCD2(-) cell line from which the PD20 RV:D2 cells were derived [40]. Both the Capan-1 cells and the PD20 cells have mutations in genes that are involved in normal DNA repair (homologous recombination and interstrand crosslink repair, respectively).

For DLD-1 and PD20 cells, the number of loci that passed filters ranged between 185 K and 260 K with an average depth of between of 56 and 62 reads per locus respectively. Only 124 K loci were called for Capan-1 cells, with an average depth of 71 reads per locus. To capture MST differences between the DNA repair proficient and DNA repair defective cell lines we first evaluated haplotypes and the presence of minor alleles for each cell line. Both DLD-1 and Capan-1 cells significantly differ with respect to haplotype distribution from DNA repair proficient cells (table 4). Capan-1 cells showed a significant decrease in heterozygotic loci, 2.1% compare to 3.3% for DNA repair proficient, which was anticipated due to the known trend for loss of heterozygosity in these cells as reported in the literature due to gene conversion in the absence of BRCA2 [42], [43]. In contrast, there was an increase (5.5%) in hetereozygotic loci in DLD-1 cells, which can potentially be attributed to increased mutation due to the MMR defects responsible for the MSI in DLD-1 cells. Surprisingly, haplotype distribution analysis at non-MST loci shows that DLD-1 cells, but not Capan-1 differ significantly from DNA repair proficient (1.8% compared to 1.2% for DLD-1 and Capan-1 respectively). This was unexpected because neither mutation mechanism (homologous recombination nor MMR) would necessarily be restricted to MST vs non-MST regions. A comparison of SNPs and INDELs in the DNA repair impaired cell lines showed Capan-1 cells significantly differed from the DNA repair proficient mean in the fraction of SNPs, with 47% and 91% for MST and non-MST loci respectively (table 5). Conversely, DLD-1 and PD20 cells were not found to be different from DNA repair proficient cell lines. For the DNA repair proficient cells the mean fraction of loci with minor alleles was 5.1% with a SD of 0.4%. Capan-1 cells showed again, a greater susceptibility to mutation with a significant increase (6.2%) in the number of loci with minor alleles (table 4). In contrast, PD20 and DLD-1 cells both show a significant decrease in loci with minor alleles, 3.1% and 3.2% respectively. This was surprising, particularly because the PD20 cells showed a decrease with respect to their corrected cell line PD20 RV:D2. Concordance of loci with minor alleles between the two related cell lines, PD20 and PD20 RV:D2, was 2.5% while discordance was 3.1%, which was significantly above chance (Pearson's X^2^). However, it was greater than the concordance between PD20 RV:D2 and MCF10A, which is to be expected since PD20 and PD20 RV:D2 are related strains (Table 3).

**Table 4:** Haplotype distribution and somatic polymorphism rate differ in DNA repair defective cell lines compared to DNA repair proficient cell lines.

	Repair Proficient		Microsatellite loci Repair impaired cell lines			Repair Proficient		Non-microsatellite loci repair impaired cell lines		
Percent (%)	Mean	SD	PD20	DLD-1	Capan-1	Mean	SD	PD20	DLD-1	Capan-1
**Homo-zyg**	96.7	0.3	97.2 #	94.5 #	97.9 #	98.9	0.2	98.8	98.2 #	99.2
**Hetero-zyg**	3.3	0.3	2.8	5.5 #	2.1 #	1.1	0.2	1.2	1.8 #	0.8
**Multi-alleles**	5.1	0.4	3.1 #	3.2 #	6.2 #	2.0	0.2	1.2 #	1.2 #	3.7 #

# significantly different p<0.01 - z-test.

**Table 5:** SNP and INDEL fractions differ in DNA repair defective cell lines compared to DNA repair proficient cells.

	Repair Proficient		Microsatellite loci Repair impaired cell lines			Repair Proficient		Non-microsatellite loci repair impaired cell lines		
Percent (%)	Mean	SD	DP20	DLD-1	Capan-1	Mean	SD	PD20	DLD-1	Capan-1
**SNPs**	36.2	3.4	35.7	36.9	47.6 #	96.9	0.2	95.4 #	94.9 #	90.8 #
**Expansions**	26.0	0.7	26.3	29.7	21.2 #	1.5	0.1	2.1 #	2.2 #	2.8 #
**Contractions**	37.9	2.9	38.0	33.3	31.2	1.6	0.2	2.5 #	2.9 #	6.4 #

# significantly different p<0.01 - z-test.

Because Capan-1 cells displayed the highest disparity in mutation rate from DNA repair proficient cell lines, including changes in SNP∶INDEL ratios, we decided to check the concordance of genotype and minor allele containing loci between them and PD20 RV:D2s (table 3). Genotype concordance for the loci that were found in both samples, was over 97.3%, even higher than when we compared PD20 RV:D2 with MCF10As. When comparing the loci with minor alleles ∼2% of the total had minor alleles in both samples (were concordant) however 12% were found to have minor alleles in only one samples, meaning discordance (table 3). Although this is strikingly different, for the PD20 RV:D2 cells to MCF10A comparison, the concordance rate is still significantly greater than expected by chance. Very similar results were obtained when Capan-1 cells were compared to MCF10A cells. These results offer additional support the hypothesis that some MST loci are more susceptible to mutations than others.

**Figure 6:**
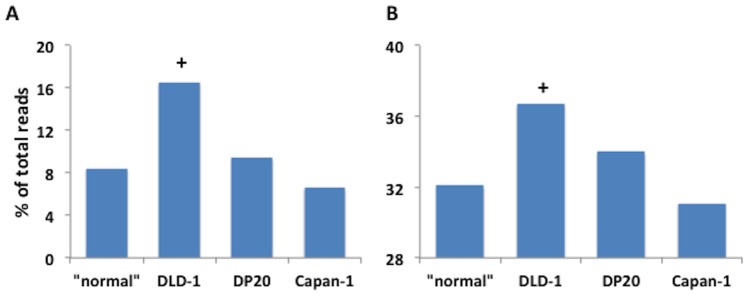
An increase in the fraction of reads substantiating the second alleles if present, and all minor alleles. The average fraction of reads representing A) all minor alleles (only for loci with minor alleles) and B) the second allele in both heterozygotic and homozygotic loci that have at least one minor allele, for DLD-1, PD20 and Capan-1 cells were compared to the average of the DNA repair proficient cell lines. The (**^+^**) denotes a significant difference from DNA repair proficient (p<0.01) with z-test.

For DLD-1 cells, the increase in heterozygotic loci coupled with the significant reduction in the number of minor alleles is counterintuitive. This suggests the possibility of a proliferation of a small number of subpopulations. If our hypothesis is correct we would anticipate two things to occur: 1) an increase the average depth of reads that define the second allele and 2) an increase in the read depth supporting minor alleles without an increase in the number. To test our hypothesis we first compared the fraction of total reads covering the second allele regardless of haplotype and reads covering only minor alleles. As depicted in figure 6, DLD-1 cells show greater than a 4% increase with respect to the DNA repair proficient average in the fractional coverage of the second allele and more than 8% increase (figure 6A and B) for the percent coverage supporting minor alleles. Both were statistically significant. Neither Capan-1 nor PD20 were found to be different from the DNA repair proficient group for either of these parameters. These results suggest a population bottleneck where only a small number of distinct subpopulations are the predominant contributors of the reads captured by the sequencer.

### SMV in exons

**Figure 7:**
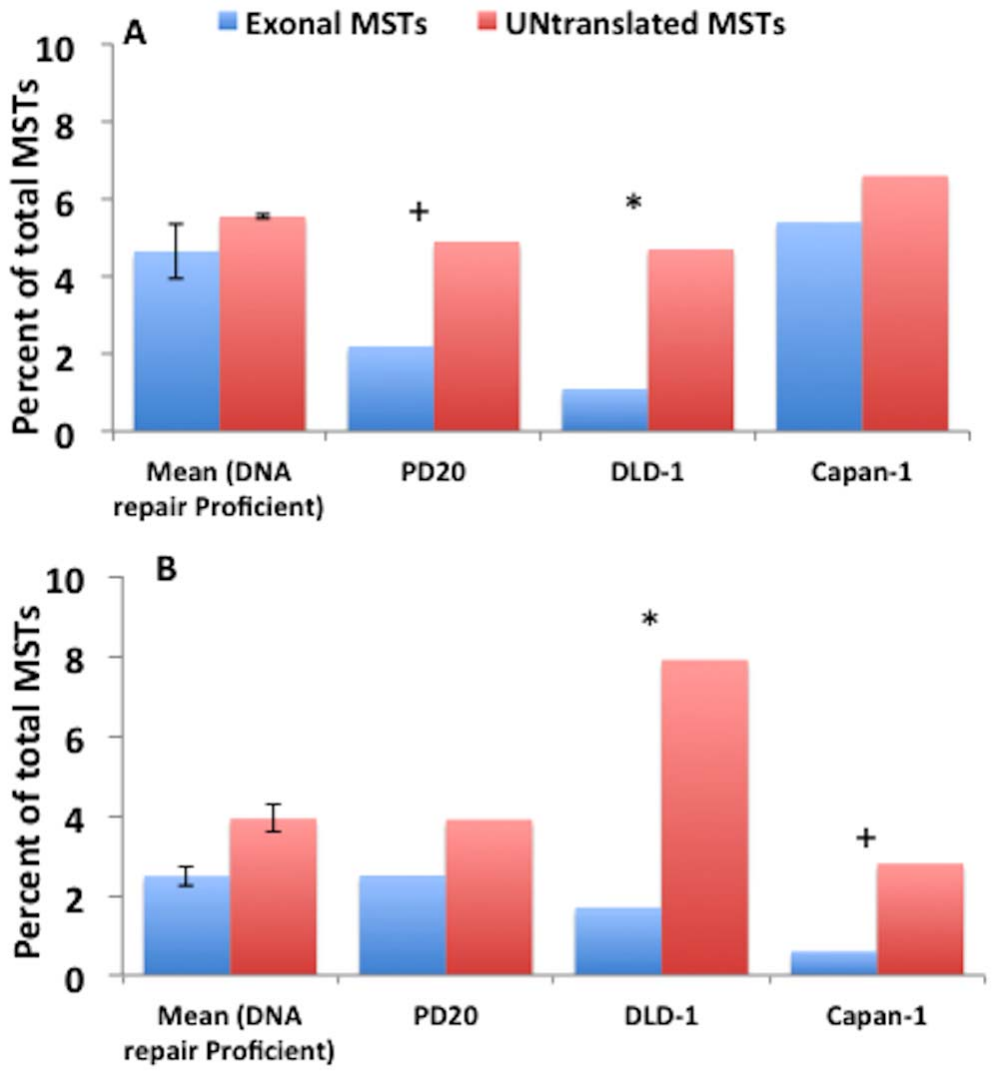
A comparison of the percent of heterozygotic loci and loci exhibiting SMV in exons and untranslated genomic regions in DNA repair proficient and impaired cell lines. A) The percent of MST loci that for which minor alleles were found and B) percent of heterozygotic MST loci, in exons and untranslated regions. Depicted in both figures are the means for the DNA repair proficient cell lines and the individual percentage for PD20, DLD-1 and capan-1 cell lines. (+) p<0.05 as compared to DNA proficient cells and (*) p<0.001 as compared to DNA proficient cells in measurement of the difference between exons and untranslated regions.

**Figure 8:**
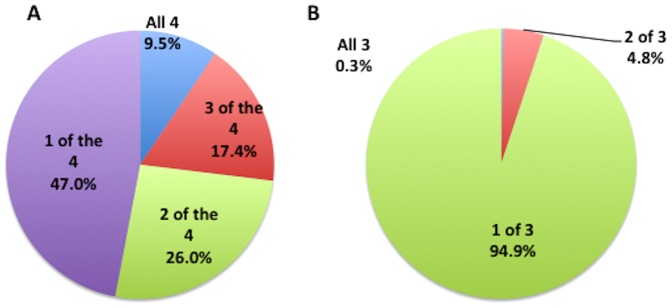
The distribution of genes that show SMV in DNA repair deficient cell lines appears random while those in the DNA repair proficient cell lines show significant similarity. The percent of genes with MSTs that with MSTs that have a minimum of 2 minor alleles in A) DNA repair proficient cell lines and B) DNA repair deficient cell lines that are found in all the or some of the sequenced samples. In figure B) the genes that are present in all three DNA repair deficient cell lines is 0.3% and the slice of the pie chart is not visible due to the small percentage.

MSTs are present ubiquitously throughout the genome and are found in over 16% of exons [1]. Although MST expansions or contractions in promoter and interexonal regions can affect transcription, mutations in exons are the most frequently implicated in downstream effects, consistent with exons being under significant selective pressure. An analysis of heterozygotic loci found that exons had significantly less heterozygotic loci, a reduction of over 1.2% compared to untranslated regions (2.4% and 3.8% respectively, figure 7A). However the difference in the fraction of loci with minor alleles in exons and untranslated regions was not significant (5.1% and 5.6%, figure 7B). In the previous sections we showed that DLD-1 cells, a strain defective in MMR, was found, unexpectedly, to have a significant reduction in the number of MST loci with minor alleles and an increase in heterozygotic loci. Based on this comparison it appears that the results are due to the increased difference between translated and untranslated regions. As shown in figure 8A, the fraction of MST loci with minor alleles in exons is 1.1% (compared to 4.7% in untranslated regions) while the fraction of loci that are heterozygotic is 1.7%, compared to 7.9% in untranslated regions (figure 8B). These results further support hypothesis that DLD-1 cells have undergone a population bottleneck.

To determine the potential genetic implications of minor allele hot spots, we focused on the analysis of genes affected, specifically we inspected genes containing MST loci found in exons that with 2 or more alleles that did not contribute to the haplotype (minor alleles). This data is provided in a spreadsheet (file S2). The spreadsheet lists the MST loci (based on the HG19 genome), gene name, cell genotype, total number of alleles, variants called and other pertinent information. Of the 2603 genes whose exons harbor minor allele containing loci found in at least one of the 4 DNA repair proficient samples sequenced 47% were found to have 2 or more minor alleles in more then one sample and 9.5% were found in all 4 samples (figure 7A). A Genome Ontology (GO) analysis of the 247 genes harboring MSTs with multiple minor alleles in all 4 samples found only a borderline (p<0.01, we use a lower p then 0.05 to compensate for the number of comparisons) significant enrichment of GOTERM categories that included transcription factors, regulators, repressors and DNA binding genes. In addition, there was no significant enrichment for any KEGG pathway categories or cataloged disorders. Conversely, of the ∼1100 minor allele harboring genes found in the DNA repair impaired cell lines, only 3 (0.27%) were found in all three cell lines while 95% are in only 1 of the three cell lines (figure 7B), which suggests this concordance pattern was primarily random. Further, no genes with multiallelic MSTs were found in all of the sequenced samples and only 18 were found in 6 of the 7 cell line samples. A KEGG pathway enrichment analysis of the minor allele harboring genes found in the DNA repair impaired cell lines suggests a pattern associated with various cancer pathways. Significant KEGG terms enriched were general cancer, colorectal cancer, myeloma, cervical cancer and cell adhesion (with p<0.001). Together, these results support the hypothesis that specific MST loci in repair proficient cells are more susceptible to somatic mutations but the genes associated with them are not associated with any specific categorized pathway. In contrast, for cells that have impairments in DNA repair pathways, somatic mutations in MSTs appear in higher frequency in loci that are specific to the DNA repair deficiency, and these mutations are implicated in disease, specifically cancer.

## Discussion

Somatic mutation can lead to subpopulations of cells carrying mutated alleles. These are examined in cancers, as tumors can be considered to contain subpopulations of cells, i.e. the tissues are not gnomically homogenous [44], [45]. Tumors usually carry an allele or set of alleles that confirm their abnormal growth. These alleles, when detected in the tumor but not parent cells, can be the basis for important clinical treatment decisions [11], [38], [45]. In cell populations with increased somatic mutation rates, like those with altered DNA repair capacity, there may be a concordant increase in subpopulation diversity. As a subpopulation propagates the mutations become more abundant, which becomes detectable in next-gen sequencing data [31], [32]. A major assumption of our analysis is that an increase in the number of alleles detected in next-gen sequencing data is reflective of an increase in cell subpopulations or somatic mutation present in the sequenced sample. In this paper we evaluate allele frequencies at MSTs in various cell populations as a quantifiable indicator of variation.

The data presented here evaluate both the standard genotype and minor alleles that are present in next-gen data to establish a baseline for SMV in DNA repair proficient cells and compare this to cells with altered DNA repair capacity. The focus on cell lines with known etiologies is to establish the viability and robustness of our approach. The results show the utility in identifying the consequences of DNA repair impairments on genomic stability. There are several major objectives/findings from this analysis including (1) complimenting genomic analysis away of matched DNA samples with in-sample quantification of variation, (2) demonstrating that DNA repair proficient cells and those with different defects in DNA repair can have different SMV profiles that may be potential markers for these defects and (3) a quantitative measure of the fraction of loci that exhibit minor alleles may be reflective of subpopulations of cells with different genomic content, potentially those cells that may contribute to tumor formation. MST instability is important in the prognosis and selection of treatment for various cancers, and better, more accurate identification methods are always being sought [10], [11].

These data demonstrate that the SNP∶INDEL ratio at MSTs can be used to distinguish between different *in-vivo* mutational mechanisms and PCR amplified genomes. Both the WGA single cell sample and the Capan-1 cell line showed an increase in SNPs compared to INDELs at MST loci, however the fractions differed greatly. This is consistent with what was expected from both nucleotide mis-incorporation errors by polymerases (WGA single cell sample) and defects in DNA repair (Capan-1). Neither DLD-1 nor PD20 cells, which are defective in MMR and interstrand cross-link repair, respectively, had a significant alteration of the ratio of SNPs∶INDELs at MST loci.

Capan-1 cells displayed a reduction of heterozygotic loci as compared to DNA repair proficient cell lines. This was expected since Capan-1 cells are a BRCA2- cells (impaired in homologous recombination) and have been shown to exhibit a loss of heterozygocity [43]. However, our analysis also indicates a significant increase in the fraction of loci with minor alleles. This could be due to two reasons: 1) Capan-1 cells are a hypotriploid with over 35 structural rearrangements (www.path.cam.ac.uk/%7epawefish/index.html) and with multiple chromosomal regions having more than three copies [46], [47]. The minor alleles in Capan-1 cells can therefore be part of the genotype rather than somatic variation. Conversely, 2) Capan-1 cells have been reported to have an extremely high rate of INDELs and SNPs, significantly higher than expected from the hyperploidy [12]. The results shown here could be due to increased mutation rate shown with this cell line [12] and further support general genomic instability in Capan-1 cells.

Unexpectedly, although DLD-1 cells are a MST unstable cell line, they did not display either of our predicted markers for increase in MST mutation rate: 1) an increase in the number of minor alleles, as was seen with Capan-1 cells, or 2) a decrease in the number heterozygotic loci and the number of minor alleles, as we found in Capan-1 and PD20 cells (table 5). Conversely, DLD-1 cells showed both a significant increase in the number of heterozygotic loci and a reduction in the fraction of loci with more than two alleles. Further, they displayed a great reduction in both the fraction of loci with minor alleles and heterozygotic loci in exons (conserved chromosomal regions). We hypothesize that this is the result of defective MMR leading to an increase in mutations that have become fixed in the population. Alternatively, this may have resulted from a bottleneck in the growth of the cell population. If this was the case, the increase in heterozygotic loci allele may be a product of a limited set of surviving cell subpopulations. If a subpopulation with an un-repaired mutation, reached a sufficient proportion of the population due to the bottleneck it would generate sufficient reads for the locus to be mistakenly called heterozygotic. This point is reinforced by the significant increase in the portion of the total number of reads covering the second allele while the fraction of loci with minor alleles and the number of minor alleles per locus are decreased. This is important to note because it suggests that we can not only distinguish between different mutational mechanisms using the minor alleles in next-gen sequencing, but may also be able to identify cells that have experienced a growth-limiting condition as we expand this work in the future.

The work presented here is a proof-of concept of an approach to assess somatic variation in MSTs using next-gen sequencing. Using this analysis we were able to establish a SMV profile in DNA repair proficient cell lines which we can use to compare to cells with potential or known alterations in DNA repair capacity to begin to evaluate exome or whole genome sequenced samples without requiring a matched genomic sample as baseline. Based on the results presented here this approach can be used to ascertain both scientifically and clinically relevant information. Scientifically, even with known mutations the consequences on the genome as a whole is still relatively unknown. Clinically, somatic variation is a measure of genomic stability and this approach might be used as an addition to current MST instability criteria.

## Supporting Information

File S1 Contains the following files: **Figure S1.** Sanger sequencing confirms the prediction of the at least 3 different alleles, in a locus found to have minor alleles in nextGen data. A) The output produced by our caller (locus is shown in the first 5 columns in line 1) predict 3 different length alleles using a minimum of 2 reads to confirm an allele. The major allele is 23 nts with 2 minor alleles, 25 and 21 nts long. B) The sequencing chromatogram. The black arrows are showing the start point of different alleles. **Figure S2.** Effects of sequencing error and the minimum number of reads required to call an allele on of the number of alleles called in sequencing data. (A) Modeling data with different error frequencies (0.5%–5%) showed an increase in loci with multiple alleles as error increased when 4 reads were minimally required to call an allele. (B) The average read depth at loci with increasing numbers of alleles using 4 confirming reads per allele for in-silico generated data using 1% and 2.5% error rate and 4 different cell lines. **Figure S3.** The distribution of MST loci showing somatic variability by chromosome for both PD20 RV:D2 samples. **Figure S4.** The distribution of MST loci showing somatic variability by chromosome, for both PD20 RV:D2, MCF10A and HEK293 cell lines. **Table S1.** In-silico model mapping and genotyping accuracy. **Table S2.** The total minor alleles sorted by MST motif length indicate that single cell exome amplification alters the distributions observed in DNA repair proficient cell lines.
(PDF)

File S2 Genomic data file.
(XLSX)
