# Supplementary material for: Exome-Wide Somatic Microsatellite Variation Is Altered in Cells with DNA Repair Deficiencies
Source: PLoS One. 2014 Nov 17;9(11):e110263. doi: 10.1371/journal.pone.0110263 (PMC4234249; doi:10.1371/journal.pone.0110263)

Table S1: In-silico model mapping and genotyping accuracy.

	In-silico error rate			
	5.0%	2.5%	1.0%	0.5%
**Total mapped reads with MSTs**	8202071	8863891	9915873	10481522
**MST call accuracy rate**	98.57%	98.53%	98.52%	98.33%
**MST accuracy in coding regions or introns**	99.23%	99.63%	99.62%	99.81%
**% correct zygosity calls**	99.96%	99.99%	99.98%	100%
**# incorrect zygosity calls (total)**	21 (58520)	3 (57204)	15 (58863)	0 (58558)

Table S2: The total minor alleles sorted by MST motif length indicate that single cell exome amplification alters the distributions observed in DNA repair proficient cell lines.

MST motif Length	PD20 RV:D2-1	PD20 RV:D2-2	MCF10A	HEK293	Single Cell
**1-nt**	65.2	66.2	55.7	62.3	12.0 #
**2-nt**	10.4	9.9	12.6	10.7	13.1
**3-nt**	8.0	7.8	11.3	8.8	55.2 #
**4-nt**	3.1	3.1	3.5	3.5	2.8
**5-nt**	7.0	7.0	8.0	7.4	13.8 #
**6-nt**	6.3	6.1	8.9	7.3	3.1

Figure S1: Sanger sequencing confirms the prediction of the at least 3 different alleles, in a locus found to have minor alleles in nextGen data. A) The output produced by our caller (locus is shown in the first 5 columns in line 1) predict 3 different length alleles using a minimum of 2 reads to confirm an allele. The major allele is 23 nts with 2 minor alleles, 25 and 21 nts long. B) The sequencing chromatogram. The black arrows are showing the start point of different alleles.

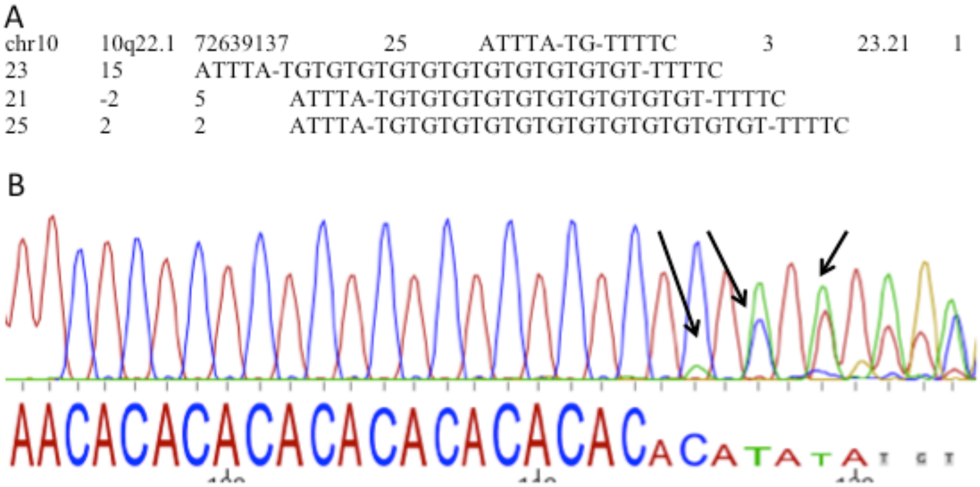

Figure S2: Effects of sequencing error and the minimum number of reads required to call an allele on of the number of alleles called in sequencing data. (A) Modeling data with different error frequencies (0.5% - 5%) showed an increase in loci with multiple alleles as error increased when 4 reads were minimally required to call an allele. (B) The average read depth at loci with increasing numbers of alleles using 4 confirming reads per allele for in-silico generated data using 1% and 2.5% error rate and 4 different cell lines.

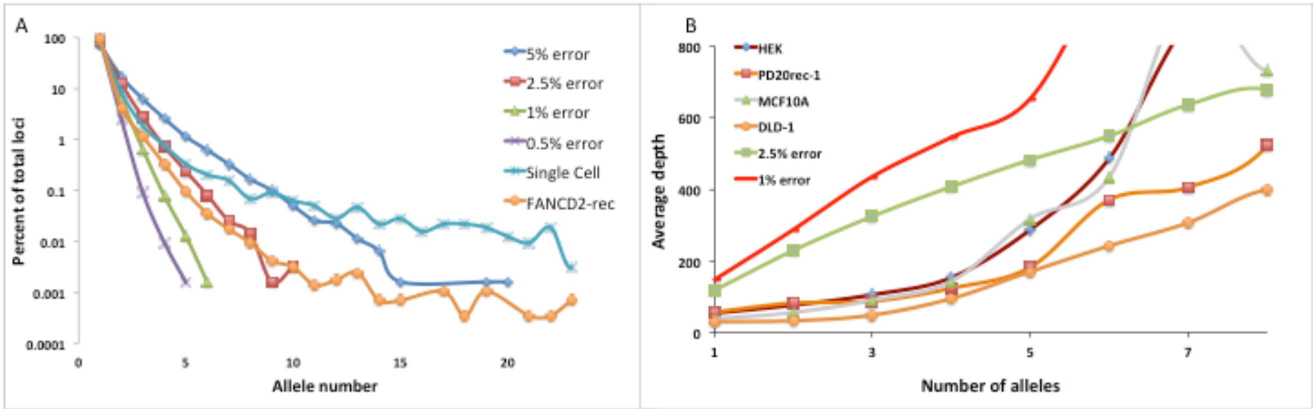

Figure S3: The distribution of MST loci showing somatic variability by chromosome for both PD20 RV:D2 samples.

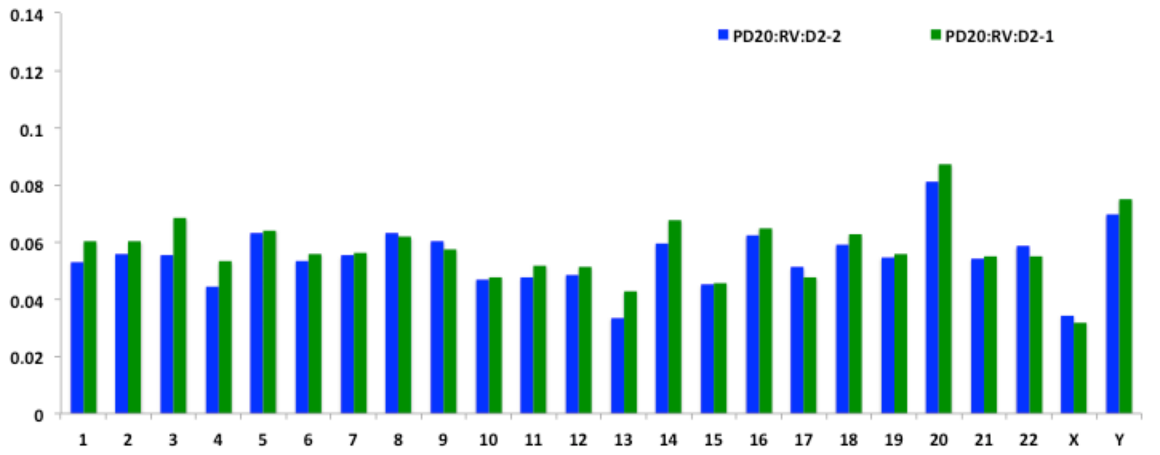

Figure S4: The distribution of MST loci showing somatic variability by chromosome, for both PD20 RV:D2, MCF10A and HEK293 cell lines.

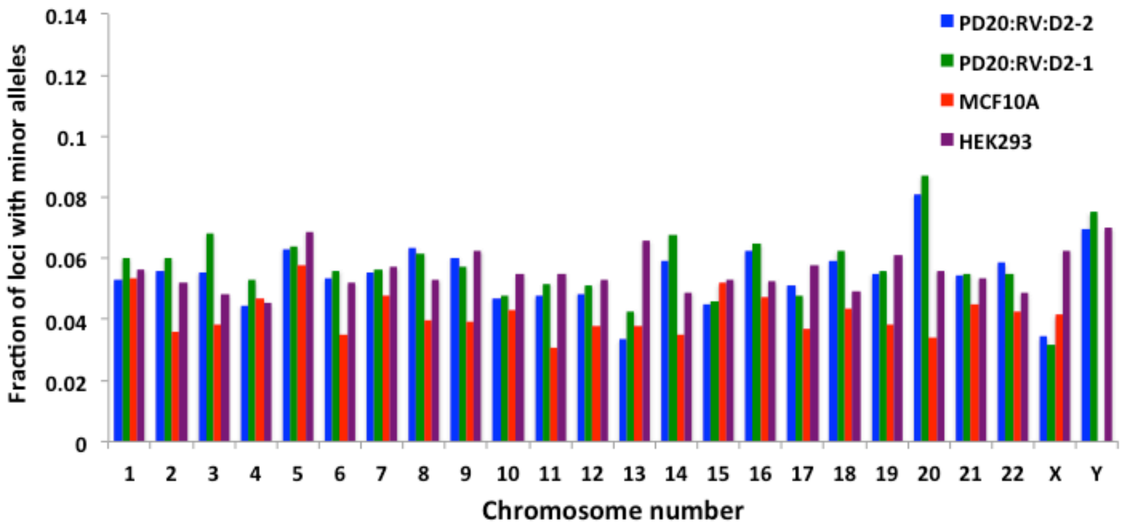

Supplement: File S1 — Contains the following files: Figure S1. Sanger sequencing confirms the prediction of the at least 3 different alleles, in a locus found to have minor alleles in nextGen data. A) The output produced by our caller (locus is shown in the first 5 columns in line 1) predict 3 different length alleles using a minimum of 2 reads to confirm an allele. The major allele is 23 nts with 2 minor alleles, 25 and 21 nts long. B) The sequencing chromatogram. The black arrows are showing the start point of different alleles. Figure S2. Effects of sequencing error and the minimum number of reads required to call an allele on of the number of alleles called in sequencing data. (A) Modeling data with different error frequencies (0.5%–5%) showed an increase in loci with multiple alleles as error increased when 4 reads were minimally required to call an allele. (B) The average read depth at loci with increasing numbers of alleles using 4 confirming reads per allele for in-silico generated data using 1% and 2.5% error rate and 4 different cell lines. Figure S3. The distribution of MST loci showing somatic variability by chromosome for both PD20 RV:D2 samples. Figure S4. The distribution of MST loci showing somatic variability by chromosome, for both PD20 RV:D2, MCF10A and HEK293 cell lines. Table S1. In-silico model mapping and genotyping accuracy. Table S2. The total minor alleles sorted by MST motif length indicate that single cell exome amplification alters the distributions observed in DNA repair proficient cell lines. (PDF) [file pone.0110263.s001.pdf]
